# Carcinogenicity assessment: “modern toxicology” considerations from experience in the evaluation of a carbon nanotube

**DOI:** 10.1093/joccuh/uiaf013

**Published:** 2025-02-17

**Authors:** Jun Kanno

**Affiliations:** Division of Cellular and Molecular Toxicology, Center for Biological Safety and Research, National Institute of Health Sciences, 3-25-26, Tonomachi, Kawasaki-ku, Kawasaki, Kanagawa 210-9501, Japan; Department of Pathology, Nissan Tamagawa Hospital, 4-8-1 Seta, Setagaya, Tokyo 158-0095, Japan; Faculty of Medicine, University of Tsukuba, 1-1-1, Tennodai, Tsukuba, Ibaraki 305-8575, Japan; National Institute for Environmental Studies, 16-2, Onokawa, Tsukuba, Ibaraki 305-8506, Japan; Systems-Biology Institute, Saisei Ikedayama Bldg. 5-10-25 Higashi Gotanda Shinagawa, Tokyo 141-0022, Japan

**Keywords:** nanomaterial, carcinogenesis, asbestos, MWCNT, modern toxicology, poison science

## Abstract

The novel properties and functions of nanomaterials have naturally alerted toxicologists to the fact that such materials may also have novel effects on the human body and other living organisms. In particular, materials with high stability or biopersistency have been shown to have a tendency to accumulate in the body, leading to chronic toxicity including carcinogenicity. However, in the early stages of toxicity research, the information is often limited to the effects of short-term exposure studies, and findings on chronic effects are very much delayed. In this context, it was exceptional that studies on multi-walled carbon nanotubes (MWCNTs) have started with the verification of their potential to induce mesothelioma. This toxicological endpoint was expected on the basis of existing knowledge of asbestos and asbestos-like fiber particles. This movement has led to the achievement of the original mission of “modern toxicology,” which is “to achieve a win-win situation where both industrial promotion and safety assurance are ensured by communicating and sharing toxicity information to developers and consumers at a stage before mass production and consumption begins, that is, before massive exposure of the general public begins.”

Inaccurate toxicity assessments of asbestos in the 1980s and 1990s allowed its spread to our living environment, which is difficult to decontaminate, and the damage continues to this day. However, the case described here could be an example of realizing the proposition that “nanomaterials, the flagship of high technology, must not repeat the same mistakes.”

## Teaser

Key points

**What is already known on this topic:** The toxicity of biopersistent particles, particulate matter, and fiber particles, especially asbestos, are known to some extent.

**What this study adds:** MWCNTs have been effectively assessed for their toxicity, particularly their carcinogenicity.

**How this study might affect research, practice, or policy:** The author proposes that toxicology is made of 2 elements, “modern toxicology” and “poison science.” The win-win situation for industry and consumers, in which both industrial promotion and safety assurance are ensured, can be effectively achieved by proper use of these 2 elements.

## 1. “Modern toxicology” hand-in-hand with “poison science”

From time immemorial, humans have consumed plants, animals, and other materials from the mountains and seas, and they have accumulated knowledge about what is safe to eat and touch; this was the beginning of the “science” of “poison.” “Poison science” is the study of the mechanisms of biological action of poisons down to the molecular level along with the compilation of the Poison List. In the process of mechanistic analysis, a wide variety of test methods have been developed.

What is “modern toxicology”? It is a scholarly system with the purpose of preventing the new products created by civilization from causing harm to civilized society. Modern civilization creates new products to make life better for everyone. However, such products sometimes do bad things to people and/or to the environment that their creators do not intend. In fact, new products always cause harm in some way.

“Modern toxicology” uses the knowledge and experience of “poison science” to identify these unintended adverse effects (eg, side effects in the case of pharmaceuticals) and provide the information about them to the creators/manufacturers and consumers so that new products can be prevented from causing harm to civilized society.

## 2. How is “modern toxicology” different from “poison science”?

In “poison science,” the targets for the research are substances that are already known to be poisonous along with the information about what kind of symptoms are induced at what dose levels. This information suggests where to start looking for their biological mechanisms. This process is similar to pharmacological studies, which look for specific medical effects at pharmacological dose levels.

In contrast, “modern toxicology” deals with substances for which we do not know what kind of adverse effects are induced at what dose levels. This means that “modern toxicology” requires a comprehensive approach to study both the “dose range” and the types of “adverse effects.” “Modern toxicology” and “poison science” are often not well distinguished because both the toxic effects of poisons and the unintended adverse effects of new products are called “toxicity.” It is important to understand that these 2 studies are different in aim and approaches; it is also important to know that both as a pair are essential for the maintenance of our civilization.

## 3. Types of victims

“Modern toxicology” is a field that comprehensively studies the interaction of xenobiotics with living organisms for the above-mentioned purpose, and its main objective is “human safety.” And there are 2 types of “humans” that need to be protected. The first is people whose face you can see, for example, yourself, the person in front of you, a family member, etc. These are people who can be identified as individuals who have developed toxic symptoms. The second is a large number of people whose faces cannot be seen individually, for example, citizens, consumers, workers, or a population of a country. A daily intake of 1 ng/kg body weight/d of aflatoxin increases the number of people dying from liver cancer by 0.1 to 3 per million (http://www.inchem.org/documents/jecfa/jecmono/v040je16.htm), which would be 10 to 300 people nationwide. These are the victims whose faces cannot be identified.

## 4. Time axis

Another important aspect emphasized in “modern toxicology” is the time axis, expressed in terms of “acute toxicity,” “chronic toxicity,” or “delayed toxicity” involving fetuses and neonates, and so on. Historically, there have been several “innovative materials” that were introduced to the world as dream new materials, swept the world, but left a negative legacy and passed away. The common features of these materials have been their biopersistency and bioaccumulation and consequently “chronic toxicity,” including carcinogenicity. Some substances have added “fetal toxicity.” In other words, the “acute toxicity” was found to be low, which led to a misperception that the overall toxicity was low, and inappropriate use of the material prevailed.

## 5. Particulate matter

Particulate matter (PM) has long been considered one of the most challenging areas of toxicology. From the perspective that industrial nanomaterials are often biopersistent, the characteristics of PM can be summarized as follows:

1) Persistent PM is often shown to be very low in toxicity when orally exposed, because it is not digestible or absorbable and is directly dumped in feces.
2) When inhaled, the size and shape of the PM particles determine how far they can reach into the respiratory tract and lungs and also how they are cleared therefrom. In some cases, they may not be cleared and may accumulate in various locations inside the lung, pleural cavity, and/or local lymph nodes.
3) When administered intravascularly, PM, as a foreign substance, may have effects on the blood coagulation system (eg, embolization, thromboembolism, induction of disseminated intravascular coagulation, etc), and direct effects on the vascular endothelium (vasoconstriction, increased permeability, etc). The effects on the immune system can be complex due to adjuvant effects and other factors. In addition, there are cases of long-term accumulation in the reticuloendothelial system, such as Thorotrast (8-10-nm thorium dioxide particles), which is known to stay there for many years.

One well-known special case of PM toxicity is mesothelioma, lung cancer, and pulmonary/pleural fibrosis caused by the “fiber particulate matter” represented by asbestos.

## 6. Asbestos toxicity

**Figure 1:**
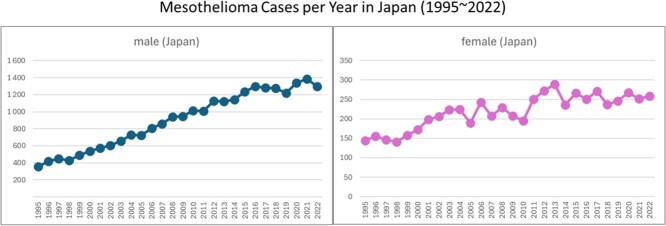
Number of mesothelioma cases in Japan from 1995 to 2022. Left: male; right: female. (Figures are drawn on the data from https://www.mhlw.go.jp/toukei/saikin/hw/jinkou/tokusyu/chuuhisyu22/index.html).

Inhaling large amounts of asbestos causes pleural thickening in almost all cases, and mesothelioma and lung adenocarcinoma occur at high rates in such patients. People who inhale small amounts of it do not develop pleural thickening (it cannot be detected by x-ray, computed tomography, or magnetic resonance imaging). In other words, it was thought that there was a “threshold” for the development of pleural thickening. However, it is strongly believed that there is no threshold for mesothelioma carcinogenesis, and this has been supported by epidemiological studies. Pleural mesothelioma is known to occur in the normal-appearing pleura without thickening. It is possible to calculate how many people per million who inhale trace amounts will develop mesothelioma, depending on the estimated inhaled amount. A lifetime risk of excess carcinogenesis of 10^–4^ is given for asbestos in the “Recommendations of the Japan Society for Occupational Health” (https://www.jstage.jst.go.jp/article/sangyoeisei/66/5/66_S24001/_pdf/-char/ja, in Japanese). Currently, mesothelioma cases in Japan are still increasing among males due to the delay in banning asbestos compared with Europe and the United States, with a total of approximately 1700 cases per year among males and females combined (Figure 1). Because mesothelioma is a rare type of malignancy and considered as a specific disease for asbestos, patients with mesothelioma can be identified, and therefore fall into the category of people whose face can be seen. In contrast, the incidence of lung cancer induced by asbestos is reported to be 2 to 6 times that of mesothelioma (https://doi.org/10.1016/j.lungcan.2024.107861). Assuming 6 times the number of lung cancer cases, the total number of asbestos-induced malignancies would rise to about 10 000 per year. Many of the asbestos lung cancer cases would be mixed in with other lung cancer cases and their faces may not always be identified. In any case, this number of patients is almost the same number obtained by calculation, ie, by multiplying Japan’s population of 100 million (10^8^) by the recommended risk level of 10^−4^. These numbers indicate how serious the contamination by asbestos is in the living environment of Japan.

## 7. Multi-walled carbon nanotubes

**Figure 2:**
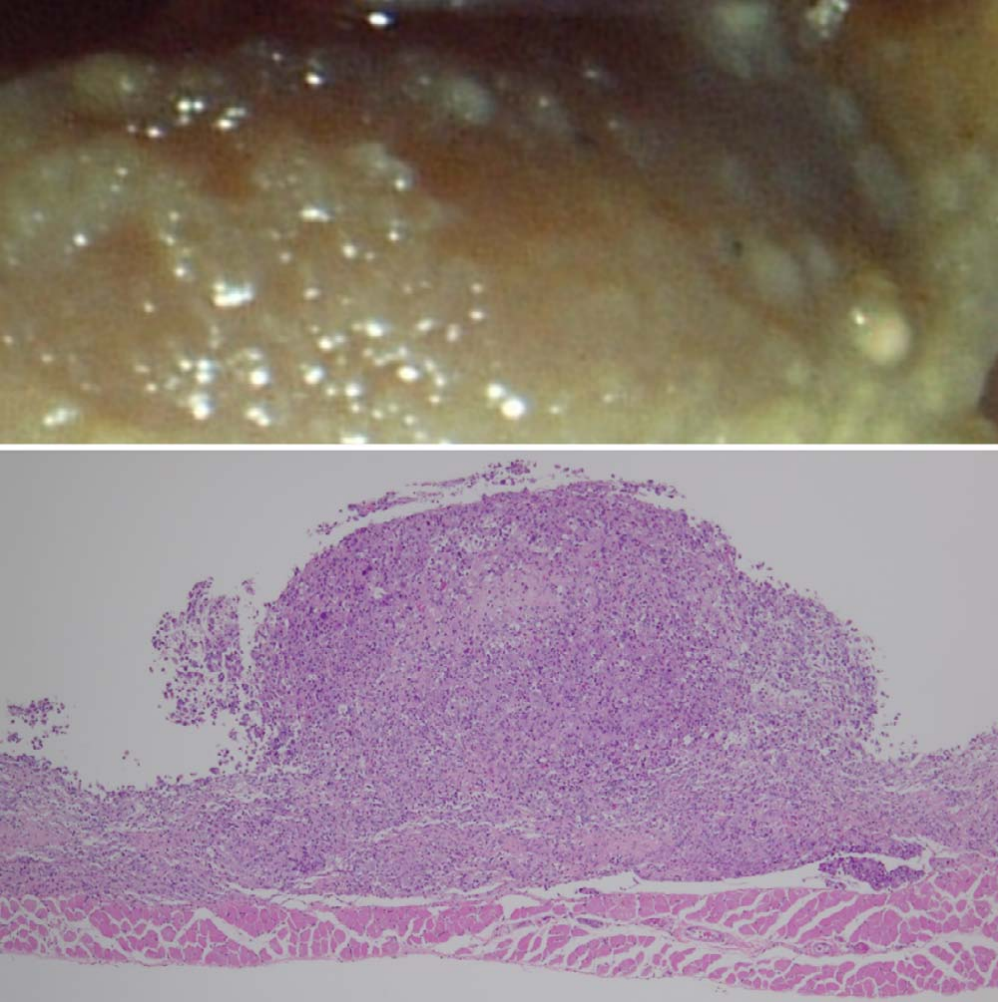
Mesothelioma induced by intraperitoneal injection of multi-walled carbon nanotubes (MWCNTs) into p53 heterozygous knockout mouse. Top: Macroscopic view of nodules of mesotheliomas on the surface of the liver of a mouse. Bottom: Light microscopic image of mesothelioma induced on the surface of the diaphragm.

The most commonly used definition of nanomaterials is “particles with at least one dimension of 100 nm or less.” Asbestos also contains rod-shaped particles with diameters of 100 nm or less. The size of fibers that have been reported to strongly induce mesothelioma is approximately 5 to 20 μm in length and about 100 nm in diameter.^1–4^ And the fact that some multi-walled carbon nanotubes (MWCNTs) contain significant fractions of fibrous particles that meet the above criteria has raised toxicological concerns. One carcinogenicity test known to be highly sensitive to asbestos is the p53 heterozygous knockout mouse (p53KO).^5^^,^^6^ This model showed that intraperitoneal administration of MWCNTs induced mesothelioma (Figure 2) in the same way as blue asbestos, or crocidolite, tested as a positive control.^7^ This test method is known as the intraperitoneal injection method. Fullerene granules (C60) are administered as a negative control. Fullerene is a carbon nanomaterial consisting of spherical primary particles with a diameter of 1 nm, and its aggregates are nonfibrous and loosely formed by van der Waals forces. An additional intraperitoneal injection study on an MWCNT demonstrating its dose–response characteristics on mesothelioma induction has also been reported.^8^

## 8. Intraperitoneal injection method

In the 1970s and 1980s, when the need arose to evaluate the carcinogenic potential of the alternative fibers to asbestos, the inhalation tests in rodents raised the issue that asbestos used as a positive control showed low carcinogenicity. It was also noted that the incidence of mesothelioma was much lower than that of lung adenocarcinoma. Therefore, instead of inhalation studies, the intraperitoneal injection method was used for that purpose because asbestos had induced peritoneal mesothelioma in 100% of the injected animals. The method was also scientifically supported to some extent by the fact that this method targets the peritoneal lining cells, ie, mesothelial cells, which are thought to be identical to the cells lining the pleural cavity.^9^ For this reason, the intraperitoneal injection method has been adopted as the first preferred method to study the mesothelioma carcinogenicity of MWCNTs worldwide.^10^

## 9. Whole-body inhalation study and intratracheal administration study

Following the initial studies by intraperitoneal injection, the positive results of the 2-year whole-body inhalation study of an MWCNT in rats were published a few years later.^11^ In this study, lung adenocarcinomas were induced at a low frequency, but mesotheliomas were not induced, similar to the trend in whole-body inhalation exposure studies of asbestos in rats, where 80% of cases are lung carcinomas and the incidence is low.^12^ As the animal facilities for the whole-body inhalation studies are very much limited in Japan, long-term intratracheal administration studies have performed, and the lung carcinogenicity and mesothelioma carcinogenicity of various nanomaterials have been reported. For carbon-based nanomaterials, biopersistent fibers ranging from rigid needle-like materials similar to crocidolite asbestos, to flexible fibrous materials similar to chrysotile asbestos that form “furballs,” are all carcinogenic,^13^ whereas nonfibrous particles, including fullerene whiskers, which are dispersed into powder in the lung, are not carcinogenic.^14^

## 10. Conclusion

If the toxicity of new materials and new products can be predicted and assessed with a high degree of accuracy as soon as their performance is known, this information can be fed back into the development strategy of manufacturers at an early stage, thereby inducing the development of products and/or their use that do not cause harm to civilized society. This flow of events should create a win-win situation between manufacturers and users. And this is the primary objective of “modern toxicology.”

If, unfortunately, the toxicity spreads to the general population, “modern toxicology” will contribute by elucidating the mechanism of toxicity to assist in the development of methods to effectively prevent exposure, including regulatory decisions, decontamination methods, methods to promote the excretion of the substance from the body, and treatment methods.

The author had opportunities to participate in the IARC Monographs on the Identification of Carcinogenic Hazards to Humans, including the 2019 revision of its Preamble.^15^ As indicated in the title of the *Journal of the National Cancer Institute* commentary, “The IARC monographs: updated procedures for modern and transparent evidence synthesis in cancer hazard identification,” the IARC had “modernized” the process of identifying carcinogenic hazards by incorporating guidance on how to use mechanistic data.^16^ In recent years, IARC monographs have tended to commend the Group 1 classification of substances under consideration on the basis of epidemiological information. In other words, it was a certification that the substance was already causing harm to people. However, originally, “cancer prevention” was the purpose of the IARC. Therefore, the original IARC success story should have been that a new substance was identified as Group 2 and never became Group 1 as a result of international responses. The changes in the latest Preamble can be interpreted, in one way, as a change toward “modern toxicology” to get closer to cancer prevention before epidemiological data and long-term animal study data are available.

Again, once persistent carcinogens have contaminated the living environment, it becomes difficult to avoid exposure, and the costs, including assistance to the victims and decontamination, will become enormous. Such a situation, which is extremely difficult to reverse, should be avoided. Therefore, it is clear that it is of great benefit to all stakeholders to link the results of hazard assessment directly to risk management and provide feedback to product developers without waiting for exposure assessment.

Unfortunately, such a cooperative linkage is not available in the current Japanese regulatory system. In the case of MWCNTs, the developers from major companies responded spontaneously to the scientific reports and refrained from using MWCNTs in products that could have openly released them into our living environment. The author believes that this action has prevented a recurrence of the asbestos incidents. Even for nanomaterials, there is no legal risk assessment or risk management system in Japan, nor are there any legal restrictions either. The government, including the Ministry of Economy, Trade and Industry, encourages self-regulation by industry. The author would like to see a “legal” regulation of nanomaterials as a reminder of “how the contamination of our living space was avoided by major companies” and as a proof that the measures taken will not be undermined over time by other actors or importers. Indeed, some website contain photographs and videos suggesting that companies in other industrial sectors who recently incorporated carbon-based fiber materials to the their new items are treating them with impunity, seemingly without recognizing that such fibers can be asbestos-like carcinogens.

If we can say that the situation with the number of victims ranging from several thousand to several tens of thousands is sufficient to be called a “devastating event,” there have been several such “events” in the past. It is still important to look back at how the initial responses to such events took place. Particularly, for the event of the methylmercury contamination in Minamata, keywords such as Hunter-Russell^17^ and Cyclator^18^ are still important for toxicologists and developers/manufacturers to look back.

In conclusion, establishment of a system that can enforce the “win-win situation” is a paradigm that should be considered in the very near future. The key factors are strengthening the process of predicting adverse events based on scientific plausibility, without waiting for exposure assessment to begin, and to systematize procedures to transfer this information to all the stakeholders. Further investment in “modern toxicology,” both in terms of human and financial resources, not only from the regulatory authorities but also from the development and manufacturing side, is highly desirable.

## Data Availability

The data that support the findings of this study are available from the corresponding author upon reasonable request.
